# Biogeography of Korea’s top predator, the yellow-throated Marten: evolutionary history and population dynamics

**DOI:** 10.1186/s12862-019-1347-x

**Published:** 2019-01-14

**Authors:** Michael Joseph Jowers, Santiago Sánchez-Ramírez, Euigeun Song, Samer Angelone, Taeyoung Choi, Inna Voloshina, Donggul Woo

**Affiliations:** 1 CIBIO/InBIO (Centro de Investigação em Biodiversidade e Recursos Genéticos), Universidade do Porto, Campus Agrario De Vairão, 4485-661 Vairão, Portugal; 2 National Institute of Ecology, 1210, Geumgang-ro, Maseo-myeon, Seocheon-gun, South Chungcheong province 33657 Republic of Korea; 3 Department of Ecology and Evolutionary Biology, University of Toronto, 25 Willcocks, Toronto, Ontario M5S 3B2 Canada; 4 Institute of Evolutionary Biology and Environmental Studies (IEU), University of Zurich, Winterthurerstrasse, 190 Zurich, Switzerland; 5 United Administration of Lazovsky State Nature Reserve and National Park “Zov tigra”, 56 Centralnaya St. Lazo, Primorsky Krai, 692980 Russia

**Keywords:** *Martes flavigula*, Korean Peninsula, Ice ages, Yellow Sea, Dispersal, Biogeography

## Abstract

**Background:**

Peninsulas often harvest high genetic diversity through repeated southward migrations of species during glacial maxima. Studies addressing within-species evolutionary responses to climate fluctuations in northeast Asia are limited compared to other regions of the world, and more so in the Korean Peninsula. In this study, we conducted the first population-level study of the yellow-throated marten, *Martes flavigula,* from the Korean Peninsula, Russian, Taiwanese and Chinese localities in a biogeographic framework using mitochondrial (*cyt-b*, *nd2*, *cr*) and nuclear gene sequencing (*ghr*).

**Results:**

Bayesian analyses revealed a rather young population, with a split from the most recent common ancestor at around 125 kya. *Martes flavigula* likely colonized the Korean Peninsula from Mainland China through the Yellow Sea twice, ca. 60 kya and 20 kya. Korean martens diversified during the Late Pleistocene with at least two dispersal events out of Korea, towards the southwest to Taiwan (ca. 80 kya) and towards the North into Russia and eastern China; most likely after the Last Glacial Maxima (ca. 20 kya). We argue that the lack of population structure and mixed populations is possibly a consequence of the high dispersal capability of the species. The Bayesian skyline plot revealed a population decline within the last 5000 years, suggesting potential negative biotic and anthropogenic effects in the area. We find that local populations are not genetically differentiated, therefore no perceptible population structure within Korea was found.

**Conclusions:**

The topography and geography of the Korean Peninsula has played a pivotal role in its colonization. Connections between the Korean Peninsula and the Mainland through sea-level drops of the Yellow Sea at times of glacial maxima and the high dispersal capability of *M. flavigula* adds to the lack of geographical structure in this species and the paraphyly of Korean lineages.

**Electronic supplementary material:**

The online version of this article (10.1186/s12862-019-1347-x) contains supplementary material, which is available to authorized users.

## Background

*Present day species* richness and *distributions* are believed to have been strongly influenced by the Last Glacial Maxima (LGM), patterns well documented throughout diverse European and North American studies [1–5]. At glacial maxima, peninsulas often have an important role, accumulating high genetic diversity as a consequence of repeated migrations or expansions from northern localities [1, 6], and, at times, levels of genetic diversity can reflect high levels of endemism at the species or subspecies levels. In contrast, the overall species evolutionary responses to climate fluctuations in northeast Asia, and more precisely in the Korean Peninsula, are not fully understood [7]. The biogeographical history of the Korean Peninsula is complex due to intermittent mainland connections caused by global sea level changes [8–14]. Ice sheet formations in North Asia resulted in refugia [15, 16] in regions such as in the Korean Peninsula, but the LGM also facilitated connectivity throughout different periods by the drainage of the Yellow Sea resulting in land-bridge formations between the mainland and the Korean Peninsula [8, 9, 17, 18].

One interesting feature of the Korean Peninsula, from a biogeographical point of view, is that it is peripheral to some widely distributed Eurasian species, and therefore likely to hold genetic signatures through peripatric speciation events [19]. However, in the peninsula the only reported endemics have limited dispersal capability and high ecological specificity. Within the terrestrial vertebrates, only salamanders (*Karsenia koreana*, *Onychodactylus koreanus*, *Hynobius quelpartensis*, *H. unisacculus*, *H. yangi*) and frogs (*Dryophytes suweonensis*, *Pelophylax chosenicus*, *Rana uenoi*) are endemic, with a complete absence of endemic reptiles or mammals. Yet, phylogeographic and population genetic studies on small mammals reveal a strong genetic structure and unique lineages within the Korean Peninsula and adjacent areas. For example, molecular work on rodents, *Apodemus peninsulae*, *A. agrarius, Myodes regulus, Tamias sibiricus* and *Crocidura shantungensis* from the Korean Peninsula, China and Russia suggest that some areas within these regions likely acted as refugia during the Pleistocene [16, 20–23]. A few studies have addressed the complexity of the Pleistocene to better propose hypotheses on mammal distribution patterns throughout the Korean Peninsula, nearby Russia, eastern China and Japan [16, 24]. Some species are thought to have used the Korean Peninsula as a land bridge for mammals to cross to Japan up to 150 ky BP before the sea started to rise and separated them for the last time [24–29]. Subsequent sea level drops in the Late Pleistocene and more recently throughout the LGM (23.5–18 cal. ka B.P.) indicate that the paleo-coastline would have connected the Korean Peninsula, southern China and Taiwan [30, 31], allowing for new migration or expansion routes, but not to Japan [32].

Throughout the Holocene, China, Taiwan, the Korean Peninsula and Russia have undergone complex environmental changes to their coastline as a consequence to cyclical sea-level fluctuations [13, 33]. The Yellow Sea was under an estuarine environment until the mid-Holocene and geological studies suggest that the present marine conditions are therefore recent [9–12, 33]. Such land connections would have opened possible recent routes for dispersal from and to the Korean Peninsula, especially for mammals with large home ranges. Altogether, the expanding and contracting coastal area contributing to the connectivity and the isolation of new islands and the peninsula is likely to have had important consequences throughout the area. Evidence of periods of connectivity by marine regression derive from recent molecular work of the Asian badger (*Meles leucurus)* [34] and *Sorex caecutiens* [35] from the Korean Peninsula and Jeju island in the South, showing limited divergence within the Peninsula and nearby localities.

Studies on larger mammals with broad home ranges such as the red fox (*Vulves vulpes*), the grey wolf (*Canis lupus*) and the raccoon dog (*Nyctereutes procyonoides)* in Korea, China and Russia have revealed a lack of geographical structure and unclear phylogeographic patterns within the region [16, 36–38]. Thus, species with high dispersal capabilities and little habitat specificity inhabiting regions with abrupt topographical changes and episodic land connectivity are expected to show little genetic differentiation over wide geographical ranges [39]. One such example is the genus *Martes*, found in Asia, Europe and North America, with species generally showing considerable distribution ranges. However, most eastern Eurasian marten species today exhibit a disjoint distribution, reflecting population fragmentation of former wider species ranges, possibly as a consequence of glaciation events throughout their range [40, 41]. Such fragmentation events may limit gene flow, eventually leading to allopatric speciation. The only species of marten present in the Korean Peninsula is the yellow-throated marten (*Martes flavigula*). The Korean Peninsula offers a unique model to assess patterns and processes, as it is part of the northern- and eastern-most distribution limit of the yellow-throated marten. This species is widely distributed throughout southern and eastern Asia, ranging from the Himalayas to southern China, the Malay Peninsula, Sunda Islands, Siberia and The Korean Peninsula [42–46]. *Martes flavigula* is believed to have originated in tropical regions and expanded to the northeast temperate areas such as Korea and Russia after the last glacial maximum (20–18 ky BP; [47, 48]). Despite their wide distribution in both tropical and temperate zones, studies on their population structure and ecology remain scant compared to other species within the genus [42, 44, 49, 50]. Phylogenetic work on *M. flavigula* has shown low genetic divergence (0.3%) between Taiwan, Russia (Primorye), and South China (Kunming), and as in other mustelid species they are thought to have had complex colonization episodes of nearby areas [51]. However, there are limited data on the population structure and population demographics of the species within the Korean Peninsula [52, 53]. Based on their wide home ranges [44], their high dispersal capability, and the presence of relatively recent land bridge connections from the Korean Peninsula to the mainland we expect the following: 1) an absence of population structure throughout the geographical areas or locally within the Korean Peninsula, 2) multiple colonization events throughout South and North China, Russia, Taiwan and the Korean Peninsula, and 3) recent Pleistocene expansion events from mainland founder populations.

## Material and methods

### Fieldwork

**Fig. 1:**
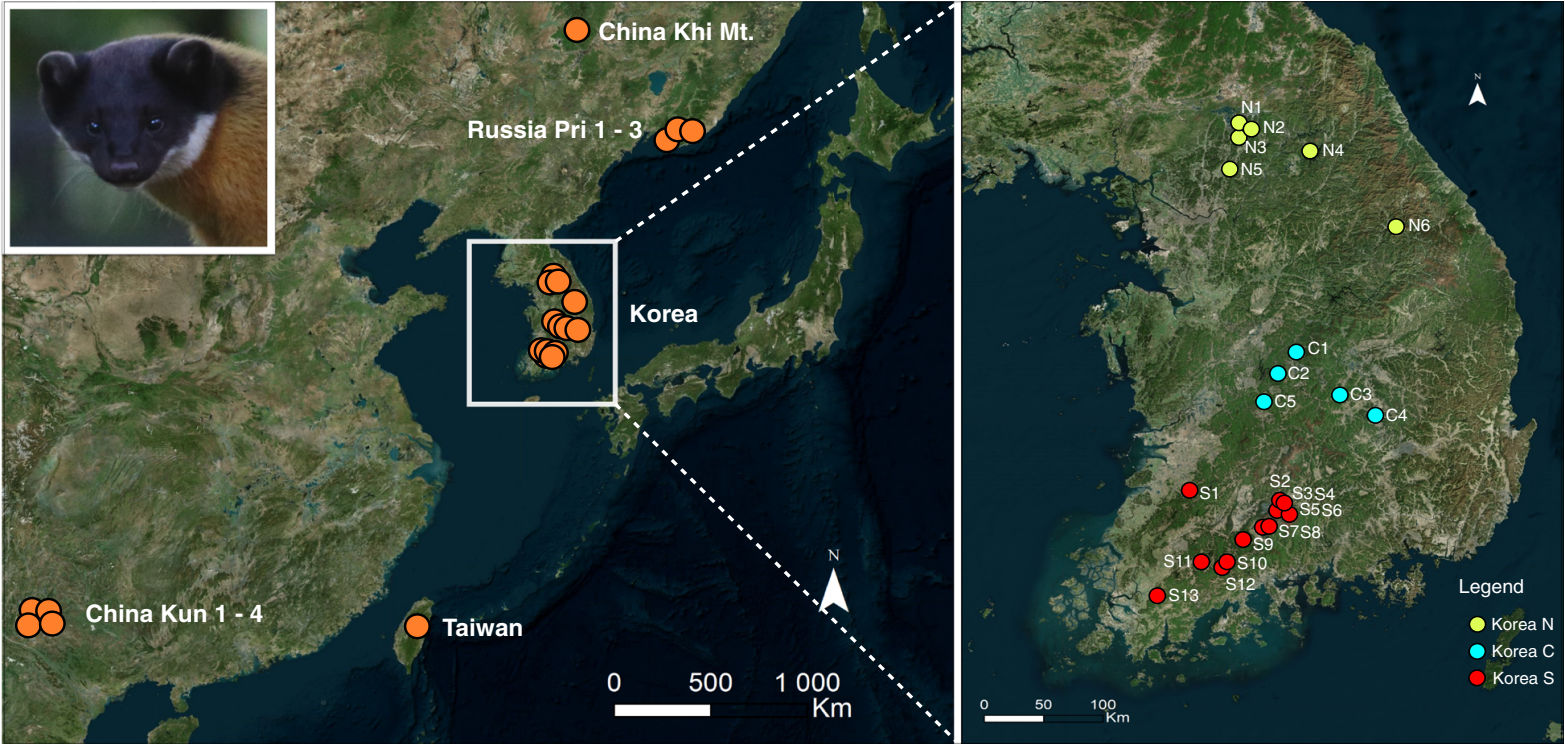
Map of all *Martes flavigula* sampled localities used in this study. Left side of the figure shows a larger map of eastern China, Taiwan, southern Russia, the Korean Peninsula and Japan. The orange dots are samples are *M. flavigula* localities. The right side of the figure represents the South Korean map and the sampling sites. Sampled sites are color coded as yellow dots (northern localities), blue dots (central localities), red dots (southern localities). Photo credit of marten head picture by Donggul Woo. Map created in QGIS v.2.18.17 (Satellite image source: Bing)

A total of 24 martens were collected throughout Korea and one individual from Russia (Fig. 1). All sampling was non-invasive as a consequence of road kills, with the only exception of a poached specimen (trapping) that died soon after attempted rescue, from which tissue samples were taken post mortem. Locality, gender and sampling are shown in Additional file 1. Tissue samples were obtained from different body parts depending on the condition (freshness) of the carcasses. Samples represent the whole distributional range of this species in South Korea, ranging proximal to the DMZ (Demilitarized zone between South and North Korea) to central and southern localities.

### Molecular work

DNA was extracted using a Qiagen DNeasy blood and tissue kit (Qiagen, Hilden, Germany) and Chelex® 100 following the instructions of the manufacturer. The targeted genes were the mitochondrial cytochrome *b* (*cyt b*), NADH dehydrogenase subunit 2 (*nd2)* and the control region (*cr)* fragments and nuclear growth hormone receptor (*ghr)*. These mitochondrial sequences were available from Genbank for *M. flavigula* from Taiwan, China and Russia [51, 54] and therefore were chosen for comparative reasons. However, such studies lacked nuclear loci and therefore the *GHR* locus was only used for population comparison among the Korean localities to assess levels of heterozygosity [55]. Polymorphic positions corresponding to heterozygous individuals in the nuclear locus were coded with IUPAC ambiguity codes and all sequences were phased using DnaSP v.5.10.1 [56]. The primers used are shown in Additional file 2. Markers were designed specific for *M. flavigula*. Internal markers were designed to resolve poor quality amplification of blood samples (Additional file 2). Templates were sequenced on both strands and the complementary reads were used to resolve rare, ambiguous base-calls in Sequencher v.4.9 (Gene Codes). Additionally, BLAST searches on gene fragments were conducted against GenBank and matches with high genetic affinity of *M. flavigula* were downloaded and included in the alignment to assess the overall phylogenetic position of Korean martens. Nine Genbank *M. flavigula* sequences from southern and eastern China, southern Russia and Taiwan were downloaded and included in the alignment (Additional file 1).

Sequences were aligned and visualized in Seaview v.4.2.11 [57] using ClustalW2 [58] default settings. We used PartitionFinder v.2 [59] to choose the optimal partitioning scheme under a greedy search [60]. Candidate outgroup species according to the literature were *M. melamprus*, *M. martes*, *M. foina* and *Gulo gulo* [61, 62]. However, after inspection of preliminary phylogenetic trees, the outgroups showed very high divergence with respect to the ingroup and we therefore run all analyses without an outgroup, rooting based only on the molecular clock model. Networks were built on Haploviewer [63] under the best tree topology as inferred in RAxML v7.0.4 [64, 65] using default settings and a GTR model. All analyses were performed through the Cyberinfrastructure for Phylogenetic Research (CIPRES [66]).

### DNA sequence data

To infer ancestry from the likely ancestral population we downloaded the only available *Martes flavigula cyt-b* sequences in Genbank from Thailand (MFL-DUZ1 Duzit Zoo, and Genbank accession AB012362MFL-CHI1, Chiang Mai Zoo, Genbank accession AB012363). However, after careful examination, the first half of the *cyt-b* sequence matched *M. melampus* and the second half *M. flavigula*. The change happened exactly after the internal *cyt-b* primers employed in their study [67] and thus indicates a possible error. Unfortunately, these two sequences have been included in numerous studies on the phylogeography of *Martes* and therefore we suspect that the chronological phylogeographic estimates, at least in part, might be inaccurate or erroneous. Thus, only the second half of the *cyt-b* (581 bp) remained usable. However, because of these issues, in addition to the unconfirmed origin (as they came from a zoo), these sequences were not included in the final analyses. Nevertheless, they were included in the MCMC (Monte Carlo Markov Chain) continuous phylogeography analysis shown in Additional file 3. One sequence of *M. flavigula* (AY882061) coming from China’s Kunming zoo was included in the analyses as it matched very closely to other martens from the Kunming Province. When geographical positioning system (GPS) coordinates were not available from published records, we used the center GPS coordinate for their alleged location.

### Bayesian coalescent and phylogeographic analyses

First, we performed statistical coalescent and phylogeographic analyses using BEAST v1.8.3 ([68]; http://beast.bio.ed.ac.uk). We inferred a mitochondrial gene tree based on a constant coalescent model, by linking all mitochondrial partitions (as they represent the same locus), but specifying separate nucleotide substitution and molecular clock models; HKY + G and strict clock for each, respectively. In order to time-calibrate the population tree, we fixed the mutation rate in *cyt-b* to 2.403 × 10^− 8^ substitutions/site/year following [69]. For the population size parameter, we used a prior lognormal distribution (in real space) with mean = 70,000 (based on a preliminary run using uninformative priors) and standard deviation = 0.5. For the prior distributions in the clock rates of *nd2* and *cr* we selected a diffuse gamma distribution (shape = 1, scale = 1 × 10^− 8^). In addition to the gene tree, we co-estimated the dispersal history using a discrete phylogeographic (ancestral state reconstruction) model also implemented in BEAST [70]. Given that our geographic sampling of populations is uneven and our state-space is low, we chose an asymmetric continuous-time rate matrix with (*n*^2^)-*n* transition-state parameters (where *n* is the number of location or states) [71]. This model assumes an asymmetric transition rate between any two states. As priors for the rates, we selected the approximate reference prior, which is specific for continuous-time Markov chain (CTMC) [72].

Secondly, we inferred changes in effective population size through time using a Bayesian Skyline Plot (BSP) model [73] with a strict clock for the *cyt-b* data and the same prior clock for the *nd2* and *cr* fragments. For both analyses, we ran two independent MCMC chains, each with 20 million states and sampling every 4000th state. Independent runs were evaluated for convergence and mixing by observing and comparing traces of each statistic and parameter in Tracer v1.6 ([74] http://beast.bio.ed.ac.uk/tracer). We considered effective sampling size (ESS) values > 200 to be good indicators of parameter mixing. The first 500 states of each run were discarded as burnin, and samples were merged using LogCombiner v1.8.3. The resulting 9000 states were summarized using TreeAnnotator v1.8.3, where a maximum-clade-credibility (MCC) tree with mean values were generated under the “-heights ca” option [75].

Thirdly, to estimate *M. flavigula* population range changes through time and ancestral population location or origin, we employed a diffusion approach of the mitochondrial sequence data through time using a continuous Bayesian phylogeographic approach [76]. We used the Cauchy (Random Walk, RRW hereafter) model with all individuals assigned to GPS coordinates with a random “jitter” with a window size of 0.5. We applied a fixed clock rate to the *cyt-b* data set and estimated rates from the prior for *nd2* and *cr*. To select for the continuous trait model we used marginal likelihood estimations (MLE) and Bayes factors (BF). MLE were calculated with path sampling (PS) and stepping stone (SS) analyses in BEAST [77] (Additional file 4). We tested for Brownian, Cauchy, Gamma and Lognormal RRW models under the strict clock models running 100 million generations, sampling every 10,000 generations. Gamma RRW ESS were low (< 50) and therefore were not estimated. For all the models tested, MLE analyses were run for 50 path steps and 100,000 generations with each step. The BF were calculated as twice the difference in MLE between alternative models, and the significance was determined if the BF value was > 10 [78]. SPREAD [79] was used to compute spatial continuous space MCC trees and viewed in Google Earth (http://earth.google.com).

Biogeographical reconstructions were performed using the statistical dispersal–vicariance analysis (S-DIVA) method [80] as implemented in RASP 2.1 [81, 82]. We used all the post-burnin trees and the MCC tree from the concatenated BEAST analyses as input of the S-DIVA analysis, sampling 1000 trees randomly. We defined 5 areas as (a) Korea; (b) Russia, (c) East China, (d) Taiwan, and (e) South China.

Tajima’s D [83], Fu’s Fs [84] and a mismatch distribution analysis [85] were estimated to examine signs of historical population expansions. Negative values of Tajima’s D and Fs can be interpreted as evidence of population expansions, and as an excess of recent mutations and reject population stasis, respectively. A diagram of frequencies of pairwise genetic differences were drawn using DnaSP v.5.10.1 [56]. One thousand bootstrap replicates were used to generate an expected distribution using a model of sudden demographic expansion [86]. Mismatch distributions are often unimodal in populations following recent population demographic and range expansion but are multimodal in samples drawn from populations at demographic equilibrium [85–88]. Based on the lack of differentiation between regions (Korean, Chinese, Russian, and Taiwanese populations), all data was considered as one population for demographic analyses.

## Results

### Alignments and markers

The nuclear *GHR* gene fragment had the lowest genetic polymorphism, with only one polymorphic site (haplotype diversity: Hd = 0.294, Nucleotide diversity per site, Pi = 0.00066), with 6 heterozygotes C or T (*n* = 1; North population, *n* = 2; center population; 3; South population), and 17 homozygotes, (n = 1, T; and *n* = 16, C). The lengths of the alignments were: 993 bp (*cyt-b*), 957 bp (*nd2*), 592 (*cr*) and 443 (*ghr*). The combined mitochondrial data set resulted in 2542 bp. For the concatenated mitochondrial data set, a total of 9 haplotypes were recovered, 5 of these from the Korean Peninsula. The *ghr* alignment recovered 2 haplotypes from the Korean Peninsula. The best partition schemes inferred by PartitionFinder, employed for the Maximum Likelihood (ML) network in Haploviewer, were: 1) *cyt-b* + *nd2* 1st codon position, *cyt-b* + *nd2* 2nd codon position, *cyt-b* 3rd codon position+*cr*, *nd2* 3rd codon position. The *ghr* network was run under no partition scheme.

### Genealogy, dispersal history and demography

**Fig. 2:**
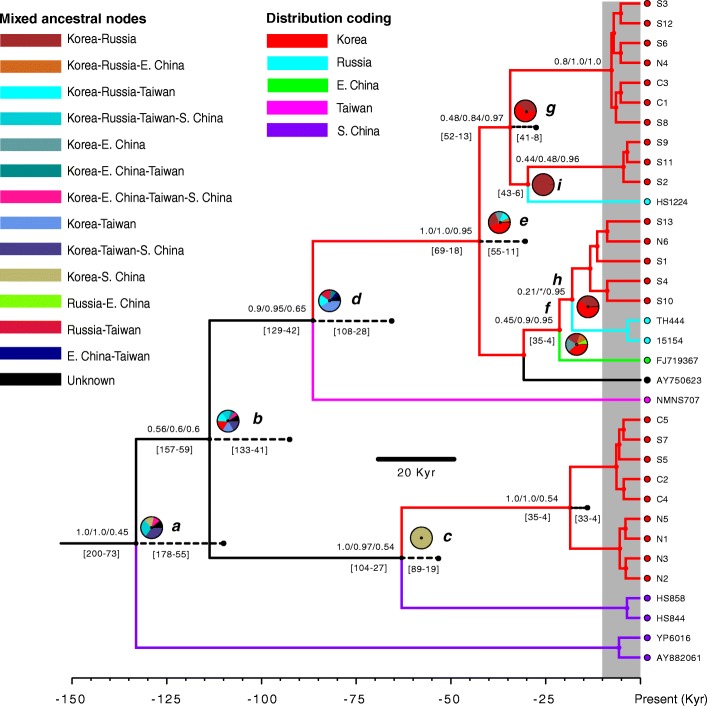
Discrete coalescent tree of *Martes flavigula*. Node circles are color coded by localities. Values by nodes are posterior probabilities recovered from the analysis. Pie charts by nodes represent results from the S-DIVA analyses of the mixed distribution nodes. Dashed black lines represent positioning of nodes from the continuous coalescent tree*.* The grey background represents the Holocene period. Values by nodes are clade posterior discrete probability, clade posterior continuous probability and state posterior discrete probability. Values under nodes are height posterior density (HPD), left is discrete and right continuous

Runs showed high Effective Sample Size (> 200), indicating adequate sampling of the posterior distribution. Both discrete and continuous MCC trees (Fig. 2), based on mitochondrial data, recovered highly similar topologies with the former showing stronger node support for the tree tips and the latter for deeper nodes. Timings were reported only from the continuous MCC tree (Additional file 5) (based on GPS coordinates) and not from the discrete analysis that relies on assigned localities (e.g., Russia vs eastern China) and can be partially subjective. The time to the most recent common ancestor (TMRCA) for these populations was estimated to be 107 kya (mean height, range 178–55 kya HPD; Highest Posterior Density) based on the continuous concatenated mitochondrial genealogy. The Korean Peninsula population was paraphyletic, and recovered two clades. The Korean Peninsula+South China clade dated ca. 50 kya (HPD; 89–19 kya) and within this clade a younger Korean clade dated 15 kya (HPD; 33–4 kya). The larger clade recovered Taiwanese, Russian, East Chinese and Korean populations. Within this clade, the Taiwanese population was sister to all other populations (63 kya, HPD; 108–28). Here, two sister clades (TMRCA; 30 kya, HPD; 55–11) were recovered, an older clade (Korean+Russian, 22 kya, HPD; 41–8) and a younger clade (Korean+Russian+East Russia, 12 kya, HPD; 27–3). The continuous MCC ancestral root (125 kya) resulted equidistant between southern China and the Korean Peninsula. Two colonization events commence at this time, one towards the West (eastern China) and a second towards the East (Korean Peninsula). The eastern lineage follows a further northern (towards the Korean Peninsula) and southern (towards Taiwan) branching event South of Jeju Island (South of the peninsula) ca. 80 kya.

**Fig. 3:**
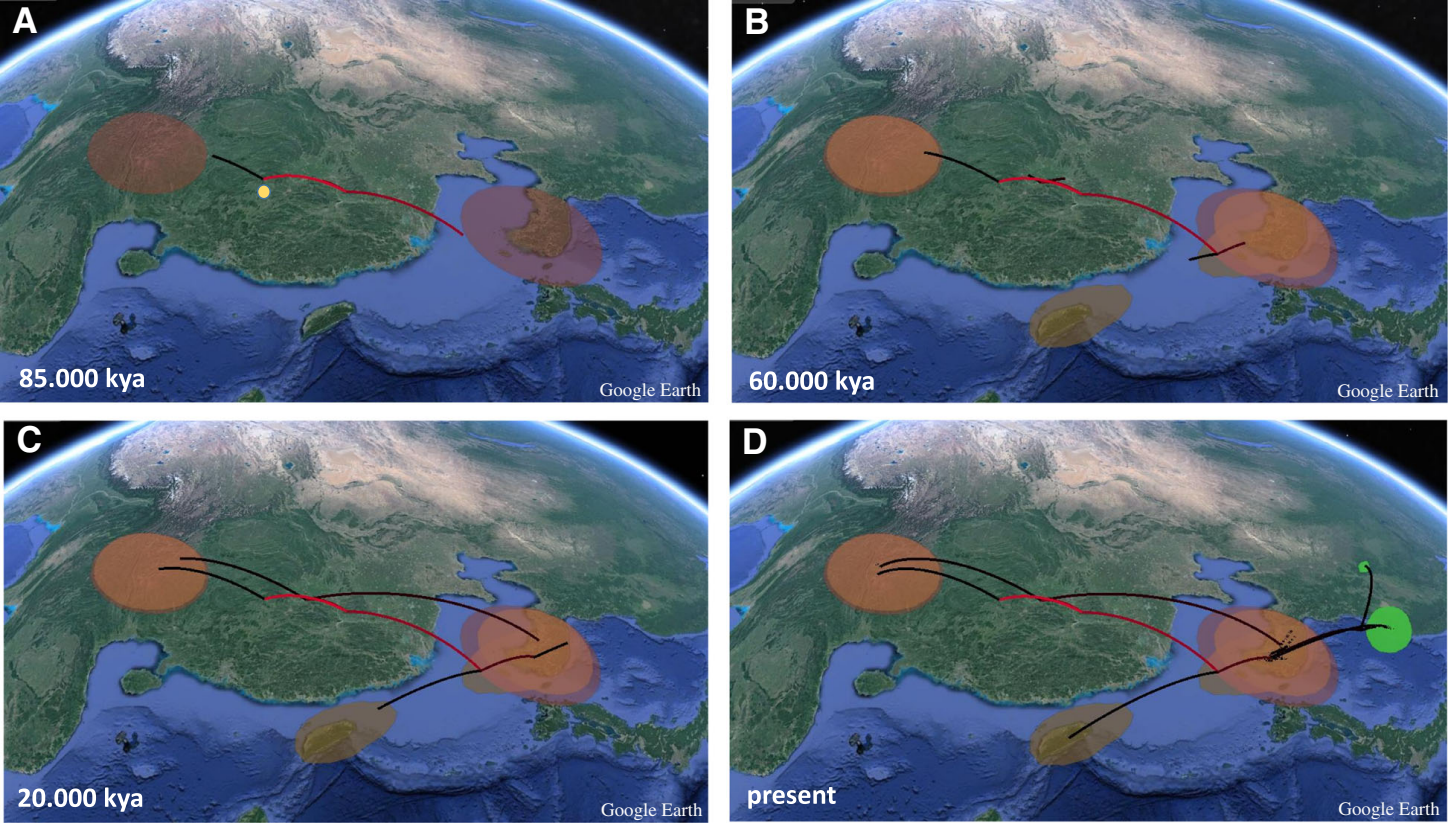
Map of the area including China, Russia, Taiwan, Korean Peninsula and Japan with Bayesian phylogeographic projections of the *Martes flavigula* population MCC tree at different time scales. Old diffusions of the MCC branches and polygons are in red and in green are recent expansions. The yellow dot represents the origin of the studied populations. **a**: population expansions up to 85 kya, **b**: up to 60 kya, **c**: up to 20 kya, **d**: up to present time. Map from Google Earth (©2018 Google)

Results suggest that the Korean Peninsula was colonized ca. 60 kya followed by Taiwan ca. 10 kya. Similarly, a second colonization event from China was inferred ca. 60 kya (Fig. 3) arriving to the peninsula ca. 20 kya. A colonization event from the Korean Peninsula towards Russia and eastern China dated to the last 10 kya (Fig. 3). The continuous *cyt-b* MCC tree that included neotropical *M. flavigula* (Additional file 3) failed to recover Thailand as ancestral to all other localities.

The greatest ancestral node uncertainty fell within older divergence estimates, as seen by weaker Bayesian posterior probabilities (Fig. 2). This was confirmed by the S-DIVA analysis that showed a limited performance to infer vicariant events in the area. Results from S-DIVA could not recover unambiguously any of the attributed assigned distribution areas as the ancestral area, as might be expected from population panmixia (Fig. 2). The highest probabilities for all nodes (a-i) were the following: node ‘a’ (Korea-Taiwan-South China 35%), node ‘b’ (three equal origins; Korea-Taiwan-South China 17%; Korea-Taiwan 17%, Korea 17%), node ‘c’ (Korea-South China 100%), node ‘d’ (Korea-Taiwan, 40%), node e’ (Korea 38.5%), node ‘f’ (Korea 38.5%), node ‘g’ (Korea 61.8%), node ‘h’ (Korea 60.5%), node ‘i’ (Korea-Russia, 98.7%). Overall the more recent divergence estimates and ancestral distributions indicated a likely Korean ancestry with diversification episodes from the Korean Peninsula into Taiwan, Russia and eastern China (Figs. 2 and 3).

**Fig. 4:**
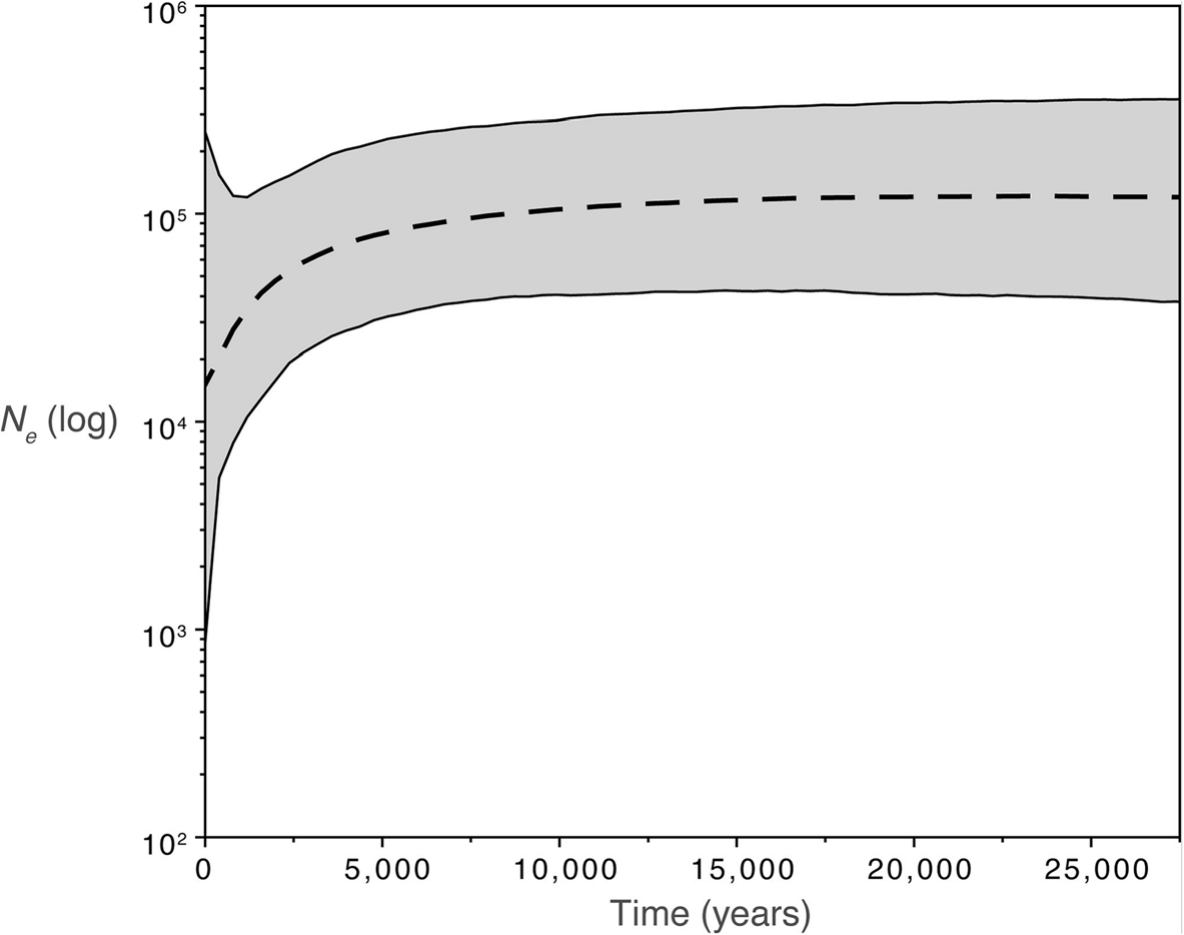
Bayesian Skyline Plot of all *Martes flavigula* (Mainland and Korean Peninsula) used in this study for the concatenated mitochondrial data set (*cyt-b*, *nd2*, *cr*)

**Fig. 5:**
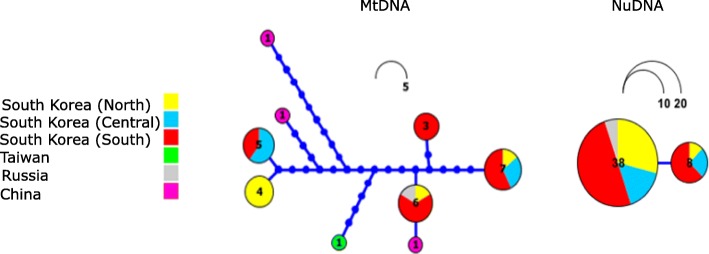
Maximum Likelihood network based on all combined mitochondrial data (*cyt-b*, *nd2*, *cr*) on the left of the figure and nuclear data (*ghr*) on the right of the figure from Korea, China, Russia and Taiwan. The network was built on Haploviewer under the best tree topology as inferred in RAxML. Colors for Korean sites correspond to colors in Fig. 1. Numbers correspond to sample frequency. Only sequences with complete data sets are included

The overall trend of the BSP suggests that the effective population size has declined over the last 5000 years, remaining constant at earlier times (Fig. 4). The multimodal mismatch distribution of the Korean samples (*n* = 24), and both Korean and mainland individuals (*n* = 29) suggests population stasis (Additional file 6). Korean samples; Fu’s F = 8.0 (significant *p* < 0.05), Tajima’s D = 2.29 (significant, p < 0.05), SSD (sum of Square Deviations = 0.10, significant *p* = 0.01), Raggedness index = 0.29 (significant, *p* = 0.001) and Mainland and Korean samples; Fu’s F = 3.9 (non-significant), Tajima’s D = − 0.2 (non-significant), SSD = 0.06 (significant p = 0.01), Raggedness index = 0.17 (significant, p = 0.001). These results confirm the observed population decline of the BSP (Fig. 4). The ML (Maximum Likelihood) RAxML network shows a lack of population structure (within northern, central or southern Korea) and with southern, eastern China, Russia and Taiwan. Within Korea, only two areas (northern and southern Korea) recovered distinct haplotypes. All other Korean haplotypes are constituted by individuals from; 1) northern, central, southern Korea, 2) central, southern Korea and 3) southern Korea and Russia (Fig. 5).

## Discussion

The topography and location of the Korean Peninsula has played a pivotal role in its colonization by many animals [7]. The repetitive connections between the Korean Peninsula and the Mainland through sea-level drops of the Yellow Sea during times of glacial maxima and the high dispersal capability of *M. flavigula* adds to the lack of geographical structure in this species and the paraphyly of the Korean population (Figs. 2, 3 and 5). *Martes flavigula* started diversifying as early as the Tarantian age (126–11.7 kya) during the Pleistocene from southern China. The ancestral root (125 kya) equidistant between the Korean Peninsula and southern China suggests at least two migrations or dispersal events towards the Korean Peninsula through the Yellow Sea. The first colonization event dates back to ca. 60 kya and a more recent arrival at around 20 kya. Although we cannot ascertain the origin of the Taiwanese population, the data and timing would support a possible Korean origin. It is likely that arrival at Taiwan as well as the event towards northern localities occurred recently, during the Holocene (ca. 9 kya; Figs. 2 and 3) following interglacial conditions and warmer periods.

The close phylogenetic affinity in *M. flavigula* from the Korean Peninsula, Russia (Primorye), East, South China (Lesser Khingan Mountains and Kumming province, respectively) and Taiwan has similarly been found in other mustelid species such as *Mustela sibirica* and *Mu. nivalis* and concurs with a scenario of recent continental marine regression during glacial periods in the area [24, 51]. Furthermore, our trees recover the same phylogenetic position as that of Taiwanese *Mu. sibirica* in relation to its counterpart populations in the Korean Peninsula, Russian and East China [24]. Four species of mustelids (including *M. flavigula)* colonized Taiwan from nearby China during the Pleistocene via land bridge connections through sea level regression at glacial periods [51]. Such colonization events support multiple episodes of migration from the Mainland, one for each species, largely in accordance with other Taiwanese fauna [89–93]. *Martes flavigula*’s estimated time of colonization (based on fossil data calibrations) between the Mainland-Taiwanese population (110 kya, HPD 190–40) lies within our estimated time range of ancestral *M. flavigula* migrations, at around the Late Pleistocene [51]. The observed mixed ancestral population origins, as inferred from the biogeographic analyses (Figs. 2 and 3) may be indicative of a recent founder population towards the East.

Genotype analyses of a *M. flavigula* population (*n* = 21) at four localities in Mountain Jiri in South Korea revealed an overall low mean relatedness and moderate genetic diversity [52]. Low kinship can often be associated with higher dispersal [94], which in mammals is attributed to resource competition avoidance and reduced levels of inbreeding [95, 96]. Furthermore, a juvenile female was reported to have moved 40 km before been road killed, again confirming their high dispersal capability [52]. These data concur with our expected lack of genetic structure throughout Korea and supports long distance movement of groups and mixing of nearby populations [97]. Similarly, other carnivores in the region such as the red fox (*Vulpes vulpes*) and the grey wolf (*Canis lupus*) with high dispersal mobility and high habitat adaptability [36, 37, 98], show unclear phylogeographic patterns and lack of population structure [99, 100]. More recently, work on red foxes in Korea, China and Russia showed admixture between Chinese and Russian populations [38]. Similarly, a lack of geographical structure in Raccoon dog (*Nyctereutes procyonoides*) populations of the Korean Peninsula has been attributed to dispersal, changing climate and geography over the past 50 kya, preceding their population expansion after the Last Glacial Maximum [16]. Furthermore, large and medium sized mammal populations from the Korean Peninsula show high genetic affinity to those from Russia, such as is the case for the Asian black bear (*Ursus thibetanus*) [101], the Siberian tiger (*Panthera tidris)* [39] and the Eurasian badger (*Meles leucurus)* [34], which suggest southward migrations from eastern Asia throughout glacial times. Contrasting findings derive from small mammals with more restricted or constrained dispersal capability, such as the Korean Field mouse (*Apodemus peninsulae*) and the Korean red-backed vole (*Myodes regulus*) from Eurasia, with Korean populations distinct from Russian and/or China, and are arguably Korean endemics [20, 21]. Other more restricted species such as the Siberian chipmunk (*Tamias sibiricus)* show strong genetic structure with northern, central and southern populations in South Korea [22]. A higher genetic differentiation correlates with earlier divergence times. For example, in the Striped field mouse (*Apodemus agrarius*), genetic divergences between populations in Russia, South Korea and central Asian populations range from 175 to 192 Kya, and between Taiwan and China from 450 to 500 kya [93]. Similarly, the divergence time of Chinese and Taiwanese populations in the Asian lesser white-toothed shrew (*Crocidura shantungensis)* dates between 192 and 397 kya. This suggests that the Korean Peninsula, Russia and Taiwan acted as refugia throughout the Pleistocene [20] for small mammals, pre-dating the arrival of *M. flavigula* to those regions.

The Yellow Sea, separating South Korea and central and northeastern China, was partly dry land throughout the last glacial period [102] and served as a land bridge between now isolated islands, and as a connective corridor at the end of the last glacial cycle [103]. Evidence of periods of connectivity by marine regression derives from recent molecular work on the Asian badger (*Meles leucurus)*, where populations from Korea (including Jeju island in the South), East Russia, Mongolia and West Siberia showed little genetic divergence between haplotypes, indicating high population dispersal [34]. Similarly, phylogenetic work on Laxmann’s shrew (*Sorex caecutiens)* from the Korean Peninsula show admixture with Asian populations but monophyly in Jeju Island [35]. The recovery of distinct mitochondrial lineages at different localities in Russia, Tsushima Islands and Taiwan, has been put forward as evidence of areas of refugia throughout the Pleistocene [24].

Further insights into the biogeography of *M. flavigula* and the timing of colonization of the Korean Peninsula can be further understood from the species distribution range and its absence in Japan, as has been shown for other species of mustelids in the region [32, 40]. Park et al. (2000) [32] argues that the shallow depths of the Korean Straight during the Last Glacial Maximum resulted in a 95% reduction of the cross-sectional area between Japan and South Korea, but both land-masses were not recently connected [32, 103, 104]. The reasoning for the non-closure lies on the paleo-Tsushima Current flowing in this area between 25 and 15 kya [32], which did not allow ice to settle in the area North of the Tsushima Island. Thus, the last of the Pleistocene connections between the Eurasian continent and Japan dates to the Late Pleistocene [28, 88, 91, 105] and therefore suggests that these land bridges occurred long before the arrival of *M. flavigula* to the Korean Peninsula, which might have happened up to 60 kya (Figs. 2 and 3). Molecular studies date such colonization events of mammals to up to 150 kya [24–28]. The Japanese weasel (*Mustela itatsi*) and the Sika deer (*Cervus nippon*) are some examples of mammals not found but that crossed over from the Peninsula to Japan before the sea started to rise [24, 28]. Thus, the presence of *M. flavigula* in Korea, in the mainland and Taiwan and absence in the Japanese islands follows patterns of topographic changes by Pleistocene climatic conditions. Similarly, successive dispersal events from the mainland to the Korean Peninsula postdate such events, as do the probable outward dispersal events from the peninsula to Taiwan (between 60 and 20 kya) and northern localities at the turn of the LGM. These data agree with LGM (ca. 23.5–18 kya) connections throughout the area, coastal China, Korea and Taiwan [14, 31]. Evidence of dispersal events derives from the lack of genetic divergence in *Mustela sibirica* mtDNA sequences between Korea and the Tsushima Islands (between South Korea and Japan) and Jeju Island, suggesting a recent land bridge connection in the area [54, 106, 107]. Similarly, the leopard cat (*P. bengalensis*) in Tsushima Islands shows close genetic affinity to the mainland and is not present in Japan [107]. Although the divergence time estimates do suggest that *M. flavigula* was already present in the Korean Peninsula at the time of the LGM sea regression, its presence in Jeju Island and Tsushima Islands has not been reported.

The causes and effects behind *M. flavigula’s* observed population decline (Fig. 4) are difficult to assess but likely reflect a combination of factors such as indirect anthropogenic impacts (e.g., through habitat disturbance and or the introduction of domestic species) and past climatic changes in the area [108, 109]. The start of *M. flavigula*’s population decline ca. 10 kya coincides with marine transgression throughout the interglacial period (~ 13–7.5 kya, [8]) and could indicate the loss of genetic diversity through northern population expansions to the mainland when new habitat became available. In addition, the more accentuated decline within the last 3 kya (Fig. 4) coincides with the first appearance of rice fields in the Korean Peninsula, which severely altered the environment through the building of rice paddies [110, 111].

## Conclusion

Here, we report on the biogeography of the yellow-throated marten from South Korean populations, Taiwan, southern and eastern China and eastern Russia. We find a recent population origin throughout its geographical range with at least two Pleistocene expansions towards the Korean Peninsula from central China. Such colonization events are likely a consequence of the proximity, topography and geography of the Korean Peninsula. The repetitive land connections between the Korean Peninsula and the Mainland through sea-level falls of the Yellow Sea at times of glacial maxima and the high dispersal capability of *M. flavigula* adds to the lack of geographical structure in this species and the paraphyly of the Korean population. In general, insufficient population sampling typically leads to a false signal of population structure. In contrast and in spite of our sample size, we do not observe structured populations, but rather a signal of high dispersal capability. These findings may be further assessed in studies with broader sample sizes and denser genetic markers.

## Additional files

Additional file 1: *Martes flavigula* codes, country of origin and sampling. (DOCX 14 kb)

Additional file 2: Primers use to amplify *Martes flavigula* gene fragments. (DOCX 12 kb)

Additional file 3: Continuous coalescent tree of *Martes flavigula* including the two short (581 bp) *cyt-b* fragments from Thailand. High posterior probability nodes are represented in red circles. Thailand is highlighted in red and South China in green. (DOCX 115 kb)

Additional file 4: Marginal likelihood estimates and Bayes factor comparison of coalescent priors for *Martes flavigula*. The asterisk (*) represents the best model selected. (DOCX 12 kb)

Additional file 5: Continuous coalescent tree of *Martes flavigula.* Posterior probability nodes are represented in red circles. (DOCX 96 kb)

Additional file 6: Mismatch distribution analyses of A) *cyt-b, B) nd2*, C) *cr* for all *Martes flavigula* individuals. (DOCX 25 kb)

## Abbreviations

BF: Bayes factors
bp: base pairs
BP: Before present
ca: circa
CIPRES: Cyberinfrastructure for Phylogenetic Research
*cr*: Control Region
CTMC: Continuous-time Markov chain
*cyt-b*: Cytochrome b
DMS: Korean Demilitarized Zone
*ghr*: Growth Hormone Receptor
GPS: Geographical positioning system
HPD: Height Posterior Density
kya: thousand years ago
LGM: Last Glacial Maxima
MCC: Monte Carlo Chain
MLE: Marginal Likelihood Estimations
*nd2*: NADH dehydrogenase subunit 2
PS: Path sampling
RRW: Repeated Random walk
S-DIVA: Statistical dispersal–vicariance analysis
SS: Stepping stone
SSD: Sum of Square Deviations
TMRCA: Time to the Most Recent Common Ancestor
