# Supplementary material for: Biogeography of Korea’s top predator, the yellow-throated Marten: evolutionary history and population dynamics
Source: BMC Evol Biol. 2019 Jan 14;19:23. doi: 10.1186/s12862-019-1347-x (PMC6332909; doi:10.1186/s12862-019-1347-x)
Supplement: Supplementary file 1 — Martes flavigula codes, country of origin and sampling. (DOCX 14 kb) [file 12862_2019_1347_MOESM1_ESM.docx]

**Additional file 1**. *Martes flavigula* codes, country of origin and sampling.

| Specimen ID | Name | Sex | Cause of Death |
| --- | --- | --- | --- |
| N1 | South Korea | No data | Wildlife Vehicle collision |
| N2 | South Korea | M | Wildlife Vehicle collision |
| N5 | South Korea | M | Wildlife Vehicle collision |
| N6 | South Korea | M | Wildlife Vehicle collision |
| N3 | South Korea | M | Wildlife Vehicle collision |
| N4 | South Korea | M | Trapping |
| C2 | South Korea | M | Wildlife Vehicle collision |
| C3 | South Korea | M | Wildlife Vehicle collision |
| C1 | South Korea | No data | Wildlife Vehicle collision |
| C4 | South Korea | M | Wildlife Vehicle collision |
| C5 | South Korea | No data | Wildlife Vehicle collision |
| S8 | South Korea | F | Wildlife Vehicle collision |
| S3 | South Korea | F | Wildlife Vehicle collision |
| S10 | South Korea | F | Wildlife Vehicle collision |
| S9 | South Korea | F | Wildlife Vehicle collision |
| S11 | South Korea | M | Wildlife Vehicle collision |
| S4 | South Korea | F | Wildlife Vehicle collision |
| S2 | South Korea | M | Wildlife Vehicle collision |
| S7 | South Korea | No data | Wildlife Vehicle collision |
| S5 | South Korea | F | Wildlife Vehicle collision |
| S1 | South Korea | No data | Wildlife Vehicle collision |
| S6 | South Korea | F | Wildlife Vehicle collision |
| S13 | South Korea | F | Wildlife Vehicle collision |
| S12 | South Korea | F | Wildlife Vehicle collision |
| 15154 | Russia, Primorye | No data | Wildlife Vehicle collision |
| TH444 | Russia, Primorye | na | na |
| HS1224 | Russia, Primorye | na | na |
| FJ719367 | East China, Kni Mt | na | na |
| HS844 | South China, kunming | na | na |
| HS858 | South China, kunming | na | na |
| YP6016 | South China, kunming | na | na |
| AY882061 | South China, kunming zoo | na | na |
| NMNS707 | Taiwan | na | na |
| AY750623 | unknown, San Diego zoo | na | na |

na- not applicable
