# Supplementary material for: Biogeography of Korea’s top predator, the yellow-throated Marten: evolutionary history and population dynamics
Source: BMC Evol Biol. 2019 Jan 14;19:23. doi: 10.1186/s12862-019-1347-x (PMC6332909; doi:10.1186/s12862-019-1347-x)
Supplement: Supplementary file 2 — Primers use to amplify Martes flavigula gene fragments. (DOCX 12 kb) [file 12862_2019_1347_MOESM2_ESM.docx]

**Additional file 2.** Primers use to amplify *Martes flavigula* gene fragments.

| Gene | Primer name | Sequence |  |
| --- | --- | --- | --- |
| *cyt-b* | Cytb-MartenF | 5'-GACCAACATTCGCAAAACTCACCCCCTGGCCA-3' | This study |
| *cyt-b* | Cytb-MartenR | 5'-GGCTAGTTGGCCGATGGTGATGAATGGA-3' | This study |
| *cyt-b* | Cytb (Marten-intR) | 5'- GAATTAGGAATAAGGCGCCTAGAATGTCC 3' | This study |
| *cyt-b* | Cytb (Marten-intF) | 5'-CAGCGTTAGCAGCAGTACATTTACTATTCC 3' | This study |
| *cyt-b* | Marten CYTB1 INT | 5´-ACC ATG AGG ACA AAT ATC CTT TTG AGG CGC AAC CG-3´ | This study |
| *cyt-b* | Marten CYTB1 INT R | 5´-CGG TTG CGC CTC AAA AGG ATA TTT GTC CTC ATG GT-3´ | This study |
| *nd2* | Nd2-MartenF | 5'-AAGCCCCCTATCCTCACCATCATCA-3' | This study |
| *nd2* | Nd2-MartenR | 5'-GGGTTAGTGGGAGTAGTATGGTTGAGA-3' | This study |
| *nd2* | ND2 (Marten-intR) | 5' GAGAGGGGAGGTAACCCTCCTAGTGAT 3' | This study |
| *nd2* | ND2 (Marten-intF) | 5' TGCCCACATGGGATGAATAATCGCTGTAAC 3' | This study |
| *nd2* | Marten ND2.1 INT | 5´-AGG CCT ATC CCC CTT CCA TTT CTG AGT GCC CGA-3´ | This study |
| *nd2* | Marten ND2.1 INT R | 5´-TCG GGC ACT CAG AAA TGG AAG GGG GAT AGG CCT-3´ | This study |
| *cr* | D-loop-MartenF | 5'-TGGTCTTGTAAACCAAAAATGG-3' | This study |
| *cr* | D-loop-MartenR | 5'-ATGTCCTGTAACCATTGACTG-3' | This study |
| *ghr* | GHR-F Mart | 5´-CCA GTT CCA GTT CCA AAG AT-3´ | Venta et al. (1996) |
| *ghr* | GHR-R Mart | 5´-TGA TTC TTC TGG TCA AGG CA-3´ | Venta et al. (1996) |

Reference: Venta PJ, Brouillette JA, Yuzbasiyan-Gurkan V, Brewer GJ. Genespecific universal mammalian sequence-tagged sites: application to the canine genome. Biochem Genet. 1996;34:321–341
