# Supplementary material for: Biogeography of Korea’s top predator, the yellow-throated Marten: evolutionary history and population dynamics
Source: BMC Evol Biol. 2019 Jan 14;19:23. doi: 10.1186/s12862-019-1347-x (PMC6332909; doi:10.1186/s12862-019-1347-x)

**Additional file 3.** Continuous coalescent tree of *Martes flavigula* including the two short (581 bp) *cyt-b* fragments from Thailand. High posterior probability nodes are represented in red circles. Thailand is highlighted in red and South China in green.


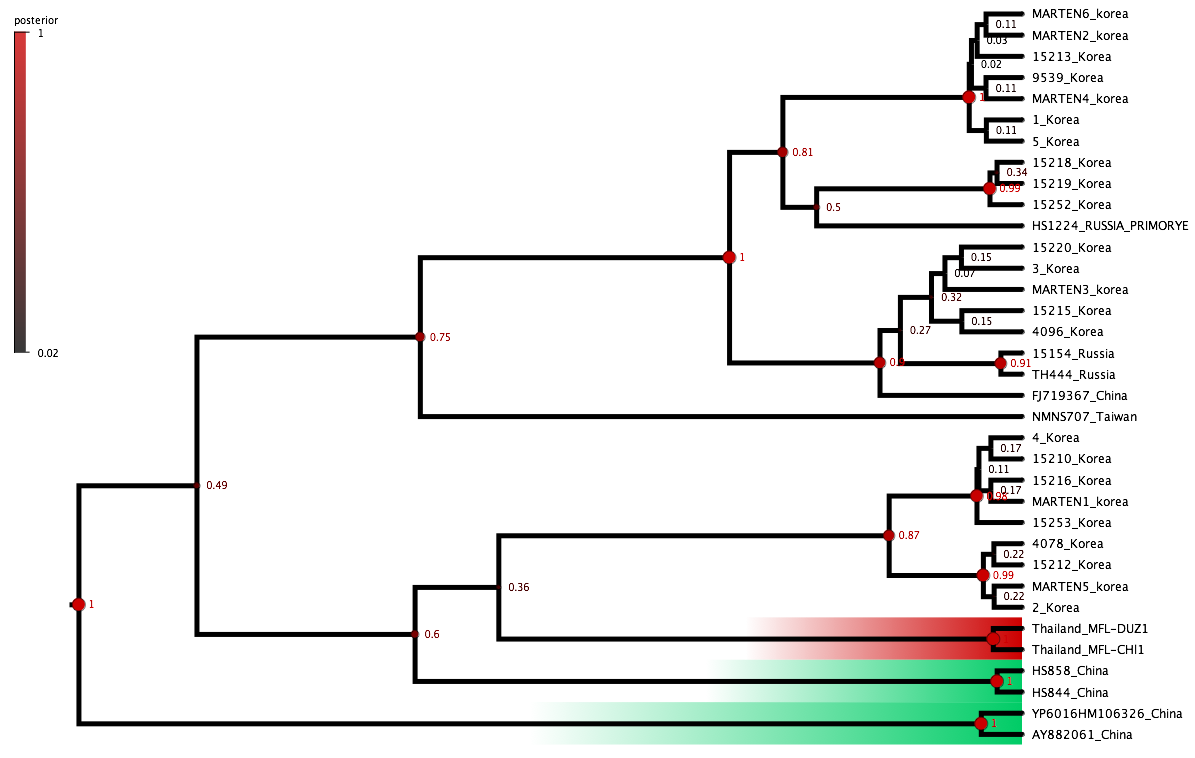

Supplement: Supplementary file 3 — Continuous coalescent tree of Martes flavigula including the two short (581 bp) cyt-b fragments from Thailand. High posterior probability nodes are represented in red circles. Thailand is highlighted in red and South China in green. (DOCX 115 kb) [file 12862_2019_1347_MOESM3_ESM.docx]
