# Supplementary material for: Biogeography of Korea’s top predator, the yellow-throated Marten: evolutionary history and population dynamics
Source: BMC Evol Biol. 2019 Jan 14;19:23. doi: 10.1186/s12862-019-1347-x (PMC6332909; doi:10.1186/s12862-019-1347-x)
Supplement: Supplementary file 4 — Marginal likelihood estimates and Bayes factor comparison of coalescent priors for Martes flavigula. The asterisk (*) represents the best model selected. (DOCX 12 kb) [file 12862_2019_1347_MOESM4_ESM.docx]

**Additional file 4**. Marginal likelihood estimates and Bayes factor comparison of coalescent priors for *Martes flavigula*. The asterisk (*) represents the best model selected.

| Models | Path Sampling (PS) | Stepping Stone (SS) | Bayes factor BS (BS) |
| --- | --- | --- | --- |
| Homogeneous Brownian | -3996.0586 | -3993.1567 | 5.80 |
|  |  |  |  |
| Cauchy RRW * | -3993.9270 | -3990.4222 | 7.00 |
|  |  |  |  |
| Lognormal RRW | -4014.0200 | -4008.9265 | 10.18 |
|  |  |  |  |
