# Supplementary material for: Biogeography of Korea’s top predator, the yellow-throated Marten: evolutionary history and population dynamics
Source: BMC Evol Biol. 2019 Jan 14;19:23. doi: 10.1186/s12862-019-1347-x (PMC6332909; doi:10.1186/s12862-019-1347-x)

**Additional file 5**. Continuous coalescent tree of *Martes flavigula.* Posterior probability nodes are represented in red circles.


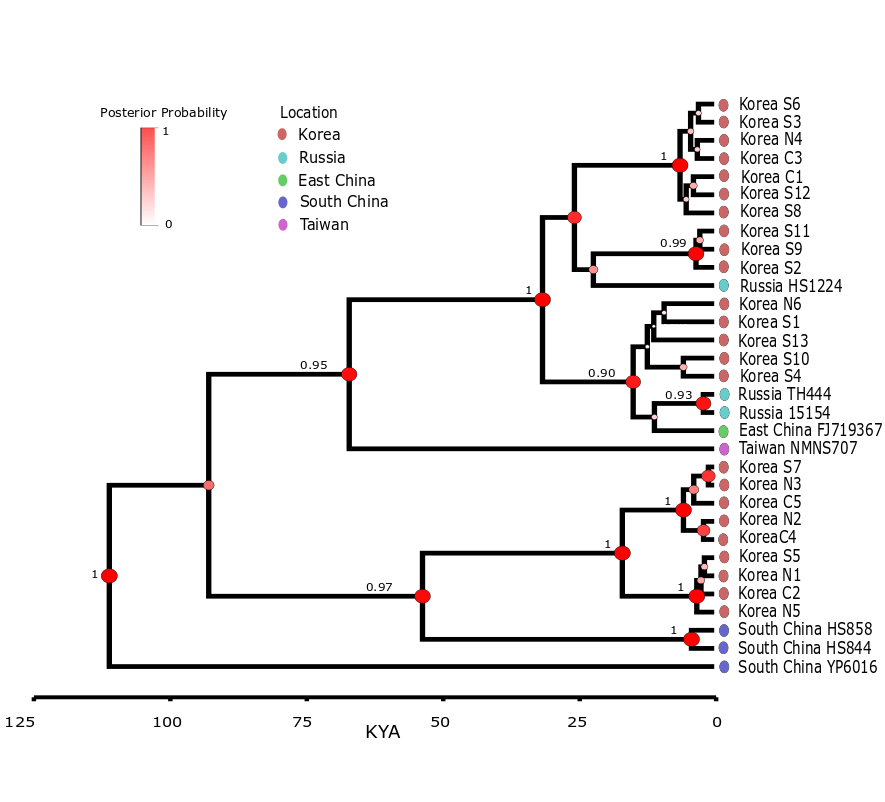

Supplement: Supplementary file 5 — Continuous coalescent tree of Martes flavigula. Posterior probability nodes are represented in red circles. (DOCX 96 kb) [file 12862_2019_1347_MOESM5_ESM.docx]
