# Supplementary material for: Biogeography of Korea’s top predator, the yellow-throated Marten: evolutionary history and population dynamics
Source: BMC Evol Biol. 2019 Jan 14;19:23. doi: 10.1186/s12862-019-1347-x (PMC6332909; doi:10.1186/s12862-019-1347-x)
Supplement: Supplementary file 6 — Mismatch distribution analyses of A) cyt-b, B) nd2, C) cr for all Martes flavigula individuals. (DOCX 25 kb) [file 12862_2019_1347_MOESM6_ESM.docx]

**Additional file 6**. Mismatch distribution analyses of A) *cyt-b, B)* *nd2*, C) *cr* for all *Martes flavigula* individuals.

1. *cyt-b*


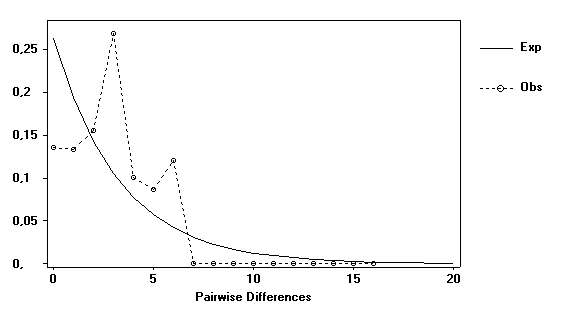


1. *nd2*

*
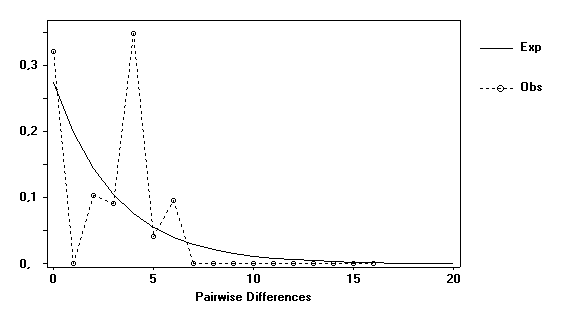
*

1. *cr*

*
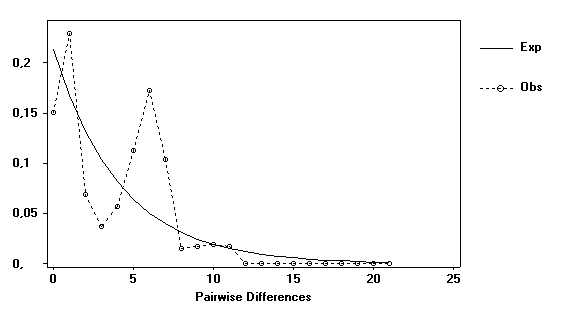
*
